# Arthroscopic Acetabular Labral Reconstruction with Fascia Lata Allograft: Clinical Outcomes at Minimum One-Year Follow-Up

**DOI:** 10.2174/1874325001611010554

**Published:** 2017-07-25

**Authors:** Ritesh Rathi, Jacek Mazek

**Affiliations:** 1 Consultant in Trauma and Orthopedics, Hinchinbrook Hospital NHS Trust, Huntingdon, Cambridge, England; 2 Consultant in Trauma and Orthopedics, Centrum Hospital Enel-med and Centre for Specialized Surgery ORTOPEDIKA, Warsaw, Poland

**Keywords:** Arthroscopic labral reconstruction, Labral tear, Hip arthroscopic surgery, Fascia lata, Allograft, Femoroacetabular impingement

## Abstract

**Background:**

The integrity of the acetabular labrum is crucial to normal biomechanics of the hip joint. Disruption of the labral seal could be detrimental to the overall nutrition of the cartilage, leading to its premature degeneration.

**Purpose:**

The aim of this study is to determine the clinical effectiveness of arthroscopic hip labral reconstruction using fascia lata allograft. The hypothesis is that labral reconstruction would provide good clinical outcomes.

**Methods:**

We retrospectively reviewed all 10 patients who underwent labral reconstruction with fascia lata allograft from January 2013 to October 2015. We assessed improvement in pain and function, complications, and subsequent surgery. The minimum follow-up was 12 months (average, 22.9 months; range, 16–36 months).

**Results:**

All patients reported subjective improvement in preoperative pain and function. The mean modified Harris hip score improved significantly from 58 (55-60) to 95 (91-98). The mean change of modified Harris hip score was 36 (31-41) and mean post-operative patient satisfaction score was 9.5(8-10). We observed no radiological progression of arthritis as well as no patient had revision procedure including total hip replacement.

**Conclusion:**

Arthroscopic labral reconstruction using a fascia lata tendon allograft is an effective and safe procedure that not only provides excellent clinical outcomes in short term but also potentially prevent continued cartilage degeneration by restoring acetabular labral seal in patients with deficient or resected labrums.

## INTRODUCTION

The integrity of the acetabular labrum is crucial to normal biomechanics of the hip joint [1]. There has been increased interest in the function of the acetabular labrum and its clinical relevance in recent times.

The acetabular labrum is a fibrocartilaginous structure that runs around the circumference of the acetabulum, forming a labral suction seal. Current evidence suggests that the hip fluid seal is important for intra-articular fluid pressurization and fluid film lubrication [2].

Consequently, disruption of the labral seal could be detrimental to the overall nutrition of the cartilage, leading to its premature degeneration [3].

The acetabular labrum enhances hip stability by effectively increasing the area of articulation between the acetabulum and the femoral head. A three-dimensional motion analysis system and cadaveric specimens with intact, vented, and torn acetabular labra, showed that 43% to 60% less force was required to distract the femur a standardized distance after venting or tearing of the labrum [4].

From the biomechanical data available, it would seem reasonable to conclude that an intact labrum provides a biomechanical advantage to the hip and every effort should be made to preserve the labrum.

The literature reviews on the results of Femoroacetabular impingement (FAI)surgery have shown the importance of labral preservation and noted that if the labrum is reattached as opposed to resected, patients have improved pain and function as determined by postoperative hip function scores [5-7].

It would seem logical to consider based on these studies that reconstruction of the labrum in the situations where the labrum was missing or beyond salvage, may improve the mechanics of the hip and lead to better pain relief and function. The purpose of this study is to determine the clinical effectiveness of arthroscopic hip labral reconstruction using fascia lata allograft.

## MATERIAL AND METHODS

A retrospective review of the 19 patients with irreparable labral tear who underwent arthroscopic acetabular labral reconstruction from January 2013 to October 2015 is carried out. For this type of retrospective study “formal consent from ethics committee is not required.”

Inclusion criteria were adult patients having undergone arthroscopic labral reconstruction with tensor fascia lata allograft (The Human Tissue Bank, Katowice,Poland) during the study period for symptomatic labral tear without advanced radiographic osteoarthritis with minimum follow-up of one year and agreed to participate in the study.

For this study, we excluded 8 patients who underwent labral reconstruction with indirect head of rectus femoris tendon autograft and are part of another report. One patient in labral reconstruction with tensor fascia lata allograft group has less than one-year follow-up is also excluded from the study. Clinical and radiographic examinations were done preoperatively, at 6 weeks and 1 year postoperatively.

### Clinical Assessments

At initial evaluation, the patient underwent physical examination that included bony and soft tissue palpation, range of motion testing, and special diagnostic tests. The special tests included the impingement test and flexion, abduction, and external rotation (FABER) test. The entire patient cohort detailed subjective questionnaire modified Harris Hip Score (mHHS) at each clinical visit (preoperative, six weeks postoperative and one year postoperative) and Patient satisfaction outcome score post-operatively once only.

### Radiological Assessments

Patients also underwent radiographic evaluation that included anteroposterior (AP) pelvis view for the examination of joint space and center edge angle, cross-table lateral view to measure alpha angle, and false profile view to determine over coverage. Acetabular over coverage was also determined by a crossover sign or posterior wall sign on AP view. Radiological progression of osteoarthritis was recorded according to Tönnis scale [8]. A magnetic resonance imaging (MRI) was completed in all patients for chondral and labral assessment.

In addition, the subjective improvement in pain, complications, revision surgeries, or conversion arthroplasties after labral reconstruction were also recorded.

### Surgical Technique

Surgical technique for Arthroscopic labral reconstruction with fascia lata allograft was described in literature [9]. Surgery was performed with the patient in the supine position on a traction table. The anterolateral portal (AP) was used for viewing using a 70° arthroscope and the mid anterior portal (MAP) was used as a working portal. Diagnostic arthroscopic surgery was performed initially for the whole joint.

Once the decision for labral reconstruction was undertaken, nonfunctional part of the labrum is removed until healthy labrum edges were achieved and the size of the defect was measured. Acetabuloplasty was then performed to prepare the bed for labral reconstruction. The graft was then prepared using fascia lata allograft with Krackow stitches to a length of approximately 2 mm longer than the measured defect length on either side.

**Fig. (1):**
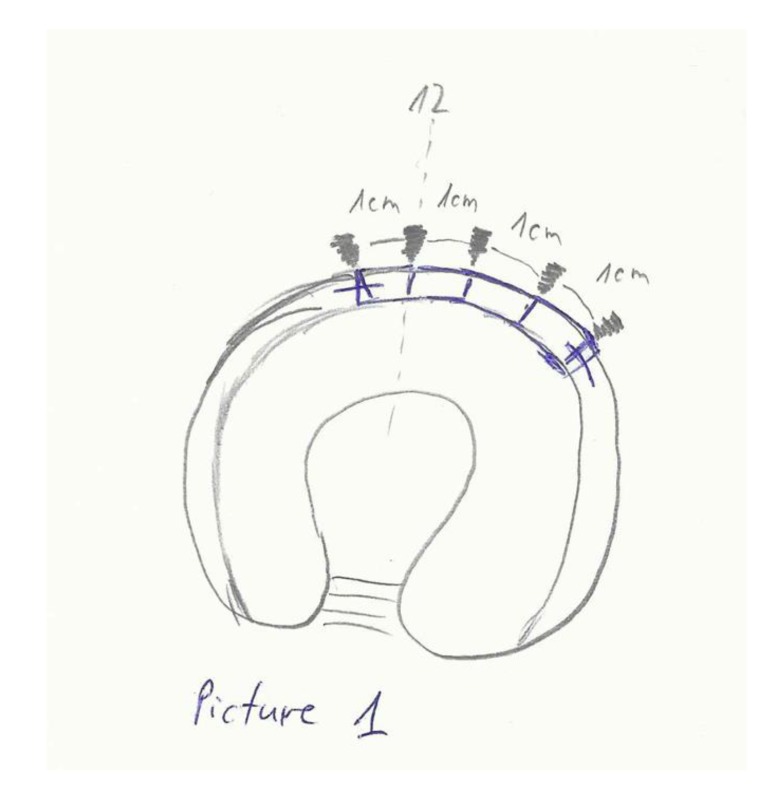
Anchor placement.

**Fig. (2):**
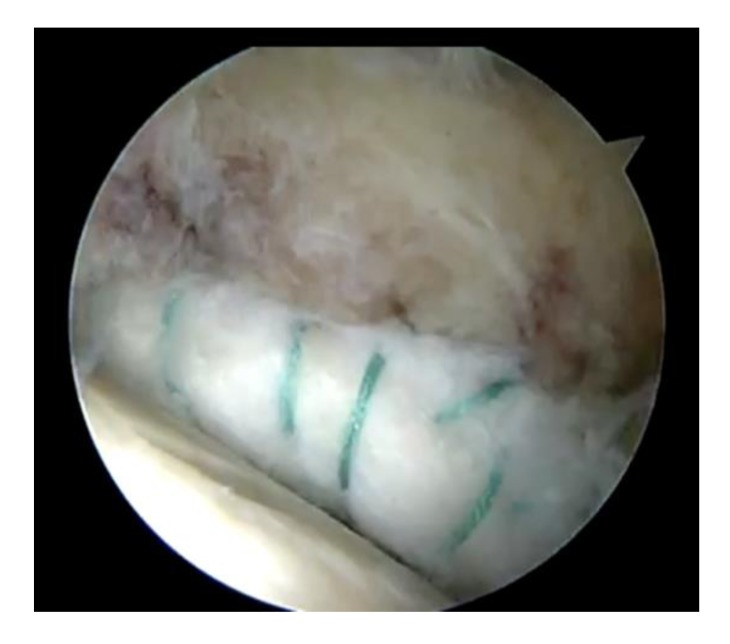
Final picture after labral reconstruction.

A third portal, the distal accessory anterior portal (DAAP) was created to drill the anchor sites at 10 mm (Fig. **1**) intervals. One of the end sutures on the end of the graft was used to lead the graft into the joint through the MAP and anchors (knotless, polyetheretherketone (PEEK) Parcus Medical™) were used to secure the graft to the acetabular rim (Fig. **2**). Gaps between the native labrum and graft were avoided.

Traction was released, and a bird’s-eye view was taken of the reconstructed labrum, demonstrating a visually appropriate initial fluid seal effect.

### Post-op Rehabilitation

Postoperatively, patients are allowed partial weight bearing on the operated extremity and 2 crutches from the first day after surgery till two weeks. Hip flexion is limited to 90 degrees and internal rotation to 20 degrees for first six weeks. Continuous passive motion machine is used for 4 weeks for 6 to 8 hours per day. Physical therapy is used to first restore passive motion, followed by active motion and, lastly, strength.

## RESULTS

There were 10 patients (all men) with a mean age of 35 years (range, 26–44 years) and with mean follow-up of 23 months (range,16–36 months). Demographics details are shown in Table **1**.

No intraoperative or postoperative complications were observed in any of the hips. We have not observed progression of osteoarthritis on plain radiographs on latest follow-up. None of the patients had undergone or scheduled for revision surgery or arthroplasty.

All the procedures were primary hip arthroscopies. Femoroplasty as well as resection of the acetabular rim was carried out in all hips with reconstruction of the labrum. A detailed overview of the intraoperative findings is given in (Table **1**). All ten patients had some degree of acetabular cartilage changes while femoral head cartilage was normal. Acetabular cartilage changes were classified according to the geographic zone method described by Ilizaliturri *et al.* [10]. Seven patients had grade II (Outerbridge classification) changes; two of them required micro fractures while five patients had debridement of the lesion. Two patients had grade III changes, one of them had microfracture of the lesion while in other case cartilage repaired with collagen gel.

**Fig. (3):**
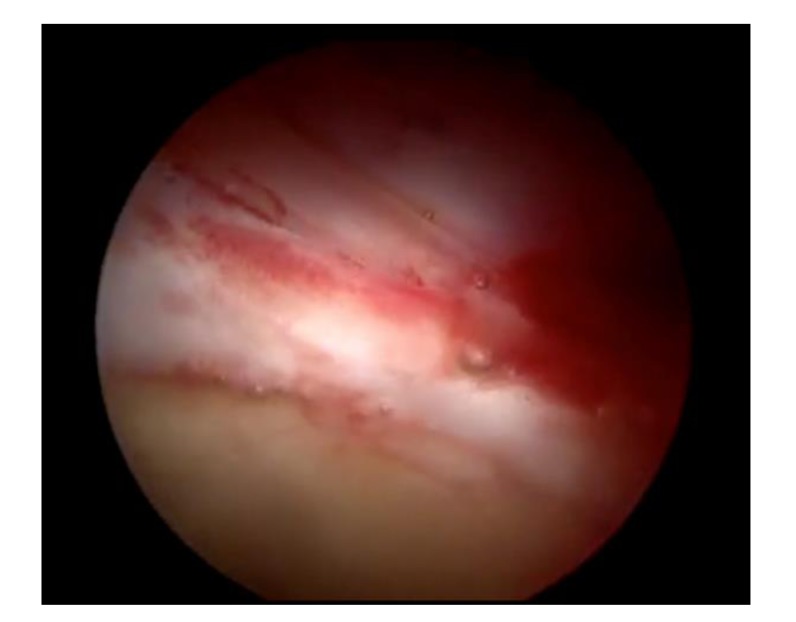
Integration of graft observed at revision surgery to address cartilage lesion.

All 10 patients reported improvement of their symptoms. Post-operative details are shown in (Table **2**). The mean mHHS was significantly better from 58 (55-60), preoperatively to 95 (91-98) at mean latest follow-up. The mean change of mHHS was 36 (31-41). All the patients are very satisfied with the outcome of the surgery with mean post-operative patient satisfaction score is 9.5(8-10). One patient form the present case series had revision hip arthroscopy to address the cartilage lesion. The intra-operative picture (Fig. **3**) confirms the integration of the graft.

## DISCUSSION

This study sought to investigate patients undergoing labral reconstruction to determine the subjective improvement in pain they experienced, the complications and reoperation rates including conversion to Total hip replacement (THR).

The present study shows that the described technique for labral reconstruction is safe, reproducible and leads to excellent subjective patient outcomes.

“The importance of the acetabular labrum has been well documented for the health and function of the hip joint.” [11] Labral tears are a significant cause of hip pain and are currently the most common indication for hip arthroscopy.

Labral lesions have been studied extensively. Although there are some studies that suggest removal of the acetabular labrum does not significantly increase the pressure or load in the acetabulum and does not predispose the hip to premature osteoarthritis, there are plenty of studies that do not support these arguments [3].

The current gold standard of treatment of labral tears is labral preservation, primarily in the form of labral repair [12]. However, in the cases where labral tears are non-repairable, labral reconstruction should be considered. The rationale is that reconstruction of the absent or insufficient labrum has the potential of restoring hip stability while recreating labral sealing properties and leads to superior clinical results [9].

Philippon *et al.* reported improvement of mean mHHS from 62 (range, 35 to 92) preoperatively to 85 (range, 53 to 100) postoperatively and median patient satisfaction was 8 out of 10 (range, 1 to 10) in a series of 95 patients who underwent labral reconstruction with Iliotibial band (ITB) autograft at a mean follow-up of 18 months [1]. Moya *et al.* reported a mean improvement of 39 points in Non arthritic hip score (NAHS), from 47(SD17.6, CI95%) to 86(SD10.5, CI95%) with 85%cases had satisfactory results in a series of 20 patients with labral reconstruction at a mean follow-up of 5.1 years [13].

The described study confirms excellent patient satisfaction and a mHHS improvement from 58 preoperatively to 93 postoperatively. These results are in agreement with the literature for joint preserving hip surgery and support the argument for labral reconstruction [1, 13, 14].

In the presented case series, we have observed no radiological progression of arthritis as well as no patient had revision procedure including total hip replacement. These findings reinforce the argument that the labral resection results in higher articular surface stresses that predispose to premature hip osteoarthritis [15].

Sierra *et al.* [16] first described the open technique while Philippon *et al.* [1] described the arthroscopic labral reconstruction. Labral reconstruction can be undertaken with either autograft or allograft. Described allografts used to labral reconstruction in the literature include; semitendinosus allograft, iliotibial band allograft, hamstring allograft and allogenic labral transplantation [17-19]. We prefer the fascia lata because it is easy to adjust the diameter of the graft and we use tabularized as they swell compare to other graft options [18].

A recently published biomechanical study of different graft choices including acetabular labrum, iliotibial band, semitendinosus, gracilis, indirect head of the rectus femoris, and anterior tibialis tendons displayed similar cyclic elongation behavior in response to simulated physiologic forces [20].

The use of allografts may restore the anatomy and labral function with no donor-site morbidity, decreased operating time, predictable graft size and quality, easier and less painful rehabilitation. The cost related to the use of allografts is higher than that of autografts, and there are still concerns about transmission of infectious diseases associated to allografts. Although with current screening, processing and sterilization techniques the risk of disease transmission with allografts is extremely low, it should not be overlooked and was taken into consideration [21].

This study has some limitations. First, it is a retrospective study with limited patient population and short follow up due to relatively recent development of the surgical technique used. Second, although our patient-reported outcome scores seem similar to those described in the literature they are not compared with control group. Third, the improvement of pain and function cannot be attributed only to the labral reconstruction; it is also related to bony corrections as well as management of cartilage lesions.

Despite these limitations, early reporting of short-term data for new procedures is prudent to publicly evaluate and discuss the role of such procedures in clinical practice and to refine their technique to maximize the benefits conferred to the patient. We encourage further higher level studies to evaluate long-term subjective and objective patient outcomes after labral reconstruction.

## CONCLUSION

Arthroscopic labral reconstruction using a fascia lata tendon allograft is an effective and safe procedure that not only provides excellent clinical outcomes in short term but also potentially prevent continued cartilage degeneration by restoring acetabular labral seal in patients with deficient or resected labrums.

## Figures and Tables

**Table 1:** Patient Demographics & Intra-operative findings.

Patient number	Age in years	Duration of Follow-up in months	Sex	Acetabular cartilage	Femoral head cartilage	Location of labral damage	Reason for labral reconstruction	Additional surgical procedure
1	32	24	M	Zone 2- 3, Grade 2	Normal	12 to 2 o’clock	Complex tear	CAM resection, Rim trimming
2	36	36	M	Zone 2- 3, Grade 2	Normal	12 to 3 o’clock	Complex tear	CAM resection, Rim trimming, micro fractures
3	28	26	M	Zone 2-3, Grade 1	Normal	11 to 3 o’clock	Complex tear	CAM resection, Rim trimming
4	42	27	M	Zone 2-3, Grade 3	Normal	9 to 12 o’clock	Complex tear & degenerative damage	CAM resection, Rim trimming, micro fractures
5	41	16	M	Zone 2-3, Grade 2	Normal	12 to 3 o’clock	Complex tear	CAM resection, Rim trimming, Debridement
6	26	21	M	Zone 2-3, Grade 2	Normal	11 to 2 o’clock	Complex tear	CAM resection, Rim trimming, Debridement
7	30	18	M	Zone 2-3, Grade 2	Normal	10 to 1 o’clock	Complex tear	CAM resection, Rim trimming, Debridement
8	33	22	M	Zone 3, Grade 2	Normal	11 to 2 o’clock	Complex tear	CAM resection, Rim trimming, Debridement
9	43	19	M	Zone 2-3, Grade 2	Normal	9 to 1 o’clock	Degenerative damage	CAM resection, Rim trimming, micro fractures
10	44	20	M	Zone 2-3, Grade 3	Normal	9 to 1 o’clock	Complex tear & degenerative damage	CAM resection, Rim trimming, cartilage repair using collagen gel

**Table 2:** Results

Patient number	Pre-op mHHS	Post-op mHHS	Improvement in mHHS	Patient satisfaction score (1-10)	Tonnis Grade (Pre-op)	Tonnis grade (Post-op)
1	59	98	39	9/10	1	1
2	58	95	37	10/10	1	1
3	58	96	39	10/10	0	0
4	60	91	31	8/10	1	1
5	56	92	36	9/10	1	1
6	58	94	36	10/10	0	0
7	55	98	43	10/10	0	0
8	60	95	35	10/10	1	1
9	57	98	41	10/10	1	1
10	60	96	36	9/10	1	1
